# Supplementary material for: Communication of preclinical emergency teams in critical situations: A nationwide study
Source: PLoS One. 2021 May 3;16(5):e0250932. doi: 10.1371/journal.pone.0250932 (PMC8092665; doi:10.1371/journal.pone.0250932)
Supplement: S1 File — (PDF) [file pone.0250932.s002.pdf]

Dear colleagues,

Within the framework of a doctoral project at the Goethe University Frankfurt am Main, we would like to ask co-workers from preclinical emergency medicine to participate in an anonymous online survey.

We would like to thank you very much for your participation.

With kind regards

Stephan Sahm (director of studies)

Matthias Zimmer (study advisor)

Daria Czarniecki (PhD student)

I agree that the data collected during the survey may be collected in encrypted, anonymous form, i.e. without naming names, e-mail or IP addresses, and may be used for scientific purposes. I am aware that it is not possible to assign the statements to myself.

Yes

No

How old are you?

How long have you been working in the rescue service?

I've been...

Male

Female

I work in the ambulance service as a...

emergency physicians

paramedics with two years training

paramedics with three years training

How great are your fears of legal, official or civil law consequences if your work would harm a patient?

In case of short-term damage (e.g. pain, indisposition)

very high high low very low

In case of a medium-term damage (e.g. extended hospital stay, rehabilitation)

very high high low very low

In case of a long-term loss (e.g. disability, death)

very high high low very low

Have you ever affected the condition of a patient through your work (e.g. pain, deterioration of condition)?

No

Yes, but the further consequences are unknown to me

Yes, short-term impairment (e.g. pain, discomfort)

Yes, medium-term impairment (e.g. extended hospital stay, rehabilitation)

Yes, long-term impairment (e.g. disability, death)

Please rate the following statements:

"In the field I would like to work with standard/guidelines"

Totally agree rather rather agree totally not agree

"Handover talks to doctors should be standardized"

Totally agree rather rather agree totally not agree

"Paramedics are good team players in the emergency services"

Totally agree rather rather agree totally not agree

"Paramedics are good team players in the ambulance business"

Totally agree rather rather agree totally not agree

"Certain team constellations are more likely to cause communication errors"

Totally agree rather rather agree totally not agree

The following statement is intended to evaluate only female employees:

"During patient care, I get less attention than my male colleagues"

totally agree rather agree rather not agree totally not agree

"Paramedics and emergency physicians could harm patients"

totally agree rather agree rather not agree totally not agree

" In action, my communication with colleagues on site is precise and effective"

totally agree rather agree rather not agree totally not agree

"I feel that my work in the emergency services is challenging.

totally agree rather agree rather not agree totally not agree

Please rate the following statements:

"When handing over patients, I forget information that was given to me"

permanently often rarely never

"When I hand over patients, I forget to pass on information"

permanently often rarely never

"I distort information during patient handovers"

permanently often rarely never

"In patient care, I forget what my colleague said to me"

permanently often rarely never

"In patient care, I address my colleague by name when I delegate a task to him"

permanently often rarely never

"In patient care, I repeat aloud the task I was told before I perform it"

permanently often rarely never

"In patient care, I announce aloud when I have completed the task assigned to me"

permanently often rarely never

"In patient care/delivery, I get interrupted if I say anything"

permanently often rarely never

"In patient care, I hear non-judgmental statements from my colleagues (e.g. make yourself useful and do this and that)"

permanently often rarely never

"When I'm very stressed, I get things mixed up"

permanently often rarely never

"When I'm very stressed, I question myself"

permanently often rarely never

"When I'm very stressed, I express myself vaguely"

permanently often rarely never

"When I'm very stressed, I use my bad tone"

permanently often rarely never

"After difficult patient care, I ask team members for feedback"

permanently often rarely never

"In routine situations misfortunes, mistakes or mistakes happen to me, if I do not actively think about the next step"

permanently often rarely never

"In stressful situations, misfortunes, blunders or mistakes happen to me"

permanently often rarely never

If you once forgot something in patient care that you were told, why did you ask me again?

There wasn't time.

There are too many tasks I have to accomplish at once

I don't want to seem unfocused.

I don't want to appear incompetent

I believe that asking is unnecessary

I have never forgotten anything

If you once forgot something you were told at the patient handover, why didn't you ask again?

There wasn't time.

There are too many tasks I have to accomplish at once

I don't want to seem unfocused.

I don't want to appear incompetent

I believe that asking is unnecessary

I have never forgotten anything

Do misunderstandings arise between you and your colleagues during patient care?

Yes, I have to inquire with colleagues exactly what they mean

permanently often rarely never I don't know if there are any misunderstandings

Yes, colleagues must inquire exactly what I mean

permanently often rarely never I don't know if there are any misunderstandings

Which of the following statements do you agree with?

Paramedics should be trained on the topic of communication (in a team/with the patient).

Emergency physicians should be trained on the topic of communication (in a team/with the patient).

Paramedics should be trained on communication (in team/with the patient) together with emergency doctors.

Which of the following statements do you agree with?

Paramedics should be trained on the topic of errors, error training or error management.

Paramedics should be trained on errors, error training or error management.

Paramedics should be trained together with emergency doctors on the topic of errors, error training or error management.

Do you think that if many small mishaps and blunders happen, this can lead to greater harm (e.g. one patient forever harm)?

Yes

No

Can you imagine that patients could be harmed by your work even if you do not intend to do so?

Yes

No

Imagine that you have made a mistake in patient care and that the patient was harmed because of it (e.g. you asked for amiodarone although you meant adrenaline). How do you feel afterwards?

I feel bad, because well trained people should not make mistakes.

I do not feel bad, because mistakes are part of everyday life.

I do not want to look bad in front of colleagues

I am ashamed

I am afraid of sanctions

Do they feel that they can learn something from other colleagues in terms of communication?

Yes, from older

Yeah, from kids the same age.

Yeah, from younger

No

Can you speak openly about misfortunes and mistakes?

No,

yes, with superiors

yes, with colleagues

yes, with my life partner, good friends, etc.

Why would you not speak openly about misfortunes and mistakes?

Fear of legal consequences.

Fear of disciplinary consequences

Shame before colleagues and superiors

No appreciation from colleagues for admissions of errors

Errors or similar are not an issue for me

I am not afraid or ashamed and I am not interested in the subject.

In your opinion, what are the general causes of poor professional communication?

Management behaviour

Work and company organisation

Character traits of the colleagues

Lack of training opportunities on this subject

Too few training courses on this subject are taken

Character traits of the own person

Other causes:

Which of these causes, which can lead to poor communication, did you personally face?

Leadership behaviour

Work and company organisation

Character traits of the colleagues

Lack of training opportunities on this subject

Too few training courses on this subject are taken

Character traits of the own person

Other causes:

What help with communication would you like to receive?

I have no need

Communication training should be offered regularly

This topic should already be an issue in vocational training

Training on this subject should be offered regularly

Supervision with a mediator should be offered regularly

Other assistance:

Please rate the following statements:

How great is your current interest in the topic of communication

very high high low very low

In which federal state are they active?
