# Supplementary material for: Communication of preclinical emergency teams in critical situations: A nationwide study
Source: PLoS One. 2021 May 3;16(5):e0250932. doi: 10.1371/journal.pone.0250932 (PMC8092665; doi:10.1371/journal.pone.0250932)
Supplement: S2 File — (PDF) [file pone.0250932.s003.pdf]

Kommunikationskompetenzen in der präklinischen Notfallmedizin

Seite 1

Kommunikationskompetenzen in der präklinischen Notfallmedizin

GOETHE UNIVERSITÄT FRANKFURT AM MAIN

Sehr geehrte Kolleginnen und Kollegen,

im Rahmen eines Promotionsvorhabens an der Goethe-Universität Frankfurt am Main möchten wir Mitarbeiter aus der präklinischen Notfallmedizin bitten, an einer anonymen Onlinebefragung teilzunehmen.

Für Ihre Teilnahme möchten wir uns herzlich bedanken.

Mit freundlichen Grüßen

Priv.-Doz. Dr. Stephan Sahm (Studienleiter)
Dr. Matthias Zimmer (Studienbetreuer)
Daria Czarniecki (Doktorandin)

Ich bin damit einverstanden, dass die im Rahmen der Befragung erhobenen Daten in verschlüsselter, anonymer Form, also ohne Namensnennung, E-Mail- oder IP-Adressenspeicherung gesammelt und zu wissenschaftlichen Zwecken ausgewertet werden. Mir ist bekannt, dass eine Zuordnung der Aussagen zu mir selbst NICHT möglich ist.

- ◯ ja
- ◯ nein

Wie alt sind Sie?

____

Wie lange arbeiten Sie bereits im Rettungsdienst?

__________ Dienstjahre

Ich bin...

- ◯ männlich
- ◯ weiblich

Ich arbeite im Rettungsdienst als...

- ◯ Notarzt/Notärztin
- ◯ Rettungsassistent/Rettungsassistentin
- ◯ Notfallsanitäter/ Notfallsanitäterin

Wie groß sind Ihre Befürchtungen vor juristischen, dienstrechtlichen oder zivilrechtlichen Folgen, wenn durch Ihre Arbeit ein Patient geschädigt werden würde?

	sehr groß	groß	gering	sehr gering
Bei einem kurzfristigen Schaden (bspw. Schmerzen, Unwohlsein)	◯	◯	◯	◯
Bei einem mittelfristigen Schaden (bspw. verlängerter Klinikaufenthalt, Reha)	◯	◯	◯	◯
Bei einem langfristigen Schaden (bspw. Invalidität, Tod)	◯	◯	◯	◯

Haben Sie durch Ihre Arbeit jemals den Zustand eines Patienten beeinträchtigt (bspw. Schmerzen, Zustandsverschlechterung)?

Mehrantworten möglich

- ☐ nein
- ☐ ja, aber die weiteren Folgen sind mir unbekannt.
- ☐ ja, kurzfristige Beeinträchtigung (bspw. Schmerzen, Unwohlsein)
- ☐ ja, mittelfristige Beeinträchtigung (bspw. verlängerter Klinikaufenthalt, Reha)
- ☐ ja, langfristige Beeinträchtigung (bspw. Invalidität, Tod)

Bitte bewerten Sie die folgenden Aussagen:

	stimme voll zu	stimme eher zu	stimme eher nicht zu	stimme nicht zu
"Im Einsatz würde ich gerne mit Standards/ Leitlinien arbeiten."				
"Die Übergabegespräche an Ärzte sollten standardisiert sein."				
"Notärzte sind gute Teamspieler im Rettungsdienst."				
"Rettungsassistenten/ Notfallsanitäter sind gute Teamspieler im Rettungsdienst."				
"Bestimmte Teamkonstellationen führen häufiger zu Kommunikationsfehlern."				
Die folgende Aussage sollen nur MitarbeiterINNEN bewerten: "Während der Patientenversorgung wird mir weniger gut zugehört als meinen männlichen Kollegen."				
"Rettungsassistenten, Rettungssanitäter, Notfallsanitäter oder Notärzte können Patienten schaden."				
"Im Einsatz ist meine Kommunikation mit den Kollegen vor Ort präzise und effektiv."				
"Meine Arbeit im Rettungsdienst empfinde ich als anspruchsvoll."				

Bitte bewerten Sie die folgenden Aussagen:

	ständig	oft	selten	nie
"Bei Patientenübergaben vergesse ich Informationen, die mir mitgeteilt wurden."				
"Bei Patientenübergaben vergesse ich, Informationen zu				

übergeben."				
"Bei Patientenübergeben verdrehe ich Informationen."	○	○	○	○
"Bei der Patientenversorgung vergesse ich, was mein Kollege/ meine Kollegin zu mir gesagt hat."	○	○	○	○
"Bei der Patientenversorgung spreche ich meinen Kollegen/ meine Kollegin mit Namen an, wenn ich eine Aufgabe an ihn/sie weitergebe."	○	○	○	○
"Bei der Patientenversorgung weiß ich stets, dass ich gemeint bin, wenn mir Aufgaben gegeben werden."	○	○	○	○
"Bei der Patientenversorgung wiederhole ich die Aufgabe laut, die man mir gesagt hat, bevor ich sie durchführe."	○	○	○	○
"Bei der Patientenversorgung sage ich laut an, wenn ich die Aufgabe erledigt habe, die mir aufgetragen wurde."	○	○	○	○
"Bei der Patientenversorgung/ Übergabe werde ich unterbrochen, wenn ich etwas sage."	○	○	○	○
"Bei der Patientenversorgung höre ich von Kollegen nicht wertschätzende Aussagen (bspw. "Machen Sie sich auch mal nützlich und tun Sie dies und das.").	○	○	○	○
"Wenn ich sehr gestresst bin, dann verwechsle ich etwas."	○	○	○	○
"Wenn ich sehr				

gestresst bin, dann verhöre ich mich."	○	○	○	○
"Wenn ich sehr gestresst bin, dann drücke ich mich unpräzise aus."	○	○	○	○
"Wenn ich sehr gestresst bin, dann kommuniziere ich immer weniger."	○	○	○	○
"Wenn ich sehr gestresst bin, dann vergreife ich mich im Ton."	○	○	○	○
"Nach einer schwierigen Patientenversorgung bitte ich die Teammitglieder um Feedback."	○	○	○	○
"In Routinesituationen passieren mir Missgeschicke, Patzer oder Fehler, wenn ich nicht aktiv über den nächsten Schritt nachdenke."	○	○	○	○
"In stressigen Situationen passieren mir Missgeschicke, Patzer oder Fehler."	○	○	○	○

Wenn Sie einmal bei der Patientenversorgung etwas vergessen haben, das Ihnen gesagt wurde, warum haben Sie dann nicht nochmal nachgefragt?

Mehrfachantworten möglich

- ☐ Dazu war keine Zeit.
- ☐ Es gibt zu viele Aufgaben, die ich gleichzeitig bewältigen muss.
- ☐ Ich will nicht unkonzentriert wirken.
- ☐ Ich will nicht inkompetent wirken.
- ☐ Ich glaube, dass Nachfragen unnötig ist.
- ☐ Ich habe noch nie etwas vergessen.

Wenn Sie einmal bei der Patientenübergabe etwas vergessen haben, das Ihnen gesagt wurde, warum haben Sie dann nicht nochmal nachgefragt?

Mehrfachantworten möglich

- [ ] Dazu war keine Zeit.
- [ ] Es gibt zu viele Aufgaben, die ich gleichzeitig bewältigen muss.
- [ ] Ich will nicht unkonzentriert wirken.
- [ ] Ich will nicht inkompetent wirken.
- [ ] Ich glaube, dass Nachfragen unnötig ist.
- [ ] Ich habe noch nie etwas vergessen.

Entstehen während der Patientenversorgung Missverständnisse zwischen Ihnen und Ihren Kollegen?

	ständig	oft	selten	nie	ich weiß nicht, ob Missverständnisse vorkommen
Ja, ich muss Kollegen fragen, was Sie genau meinen.	○	○	○	○	○
Ja, Kollegen müssen mich fragen, was ich genau meine.	○	○	○	○	○

Welchen der folgenden Aussagen stimmen Sie zu?

Mehrfachantworten möglich

- [ ] Rettungsassistenten/ Notfallsanitäter sollten zum Thema Kommunikation (im Team/ mit dem Patienten/ in Übergaben) ausgebildet werden.
- [ ] Notärzte sollten zum Thema Kommunikation (im Team/ mit dem Patienten/ in Übergaben) ausgebildet werden.
- [ ] Rettungsassistenten/ Notfallsanitäter sollten gemeinsam mit Notärzten zum Thema Kommunikation (im Team/ mit dem Patienten/ in Übergaben) ausgebildet werden.

Welchen der folgenden Aussagen stimmen Sie zu?

Mehrfachantworten möglich

- [ ] Rettungsassistenten/ Notfallsanitäter sollten zum Thema Fehler, Fehlertraining oder Fehlermanagement ausgebildet werden.
- [ ] Notärzte sollten zum Thema Fehler, Fehlertraining oder Fehlermanagement ausgebildet werden.
- [ ] Rettungsassistenten/ Notfallsanitäter sollten gemeinsam mit Notärzten zum Thema Fehler, Fehlertraining oder Fehlermanagement ausgebildet werden.

Glauben Sie, dass wenn viele kleine Missgeschicke und Patzer geschehen, dies dazu führen kann, dass ein größerer Schaden entsteht (bspw. ein Patient Schaden nimmt)?

- ○ ja
- ○ nein

Können Sie sich vorstellen, dass Patienten durch Ihre Arbeit geschädigt werden könnten, obwohl Sie dies nicht beabsichtigen?

- ○ ja
- ○ nein

Stellen Sie sich vor, Sie haben sich bei der Patientenversorgung versprochen und es kam deswegen zu einem Schaden für den Patienten (bspw. haben Sie Amiodaron verlangt, obwohl Sie Adrenalin meinten).
Wie geht es Ihnen danach?

Mehrfachantworten möglich

- [ ] Ich fühle mich schlecht, denn gut ausgebildete Kräfte sollten keine Fehler machen.
- [ ] Ich finde das nicht schlimm, weil Fehler zum Alltag gehören.
- [ ] Ich möchte nicht schlecht vor Kollegen dastehen.
- [ ] Ich schäme mich.
- [ ] Ich habe Angst vor Sanktionen.

Sind Sie der Ansicht, dass Sie von anderen Kollegen in Sachen Kommunikation etwas lernen können?

Mehrfachantworten möglich

- ☐ ja, von älteren
- ☐ ja, von gleichaltrigen
- ☐ ja, von jüngeren
- ☐ nein

Können Sie offen über Missgeschicke und Fehler sprechen?

Mehrfachantworten möglich

- ☐ nein
- ☐ ja, mit Vorgesetzten
- ☐ ja, mit Kollegen
- ☐ ja, mit meinem Lebenspartner, guten Freunden usw.

Warum würden Sie nicht offen über Missgeschicke und Fehler sprechen?

Mehrfachantworten möglich

- ☐ Angst vor juristischen Folgen
- ☐ Angst vor dienstrechtlichen Folgen
- ☐ Scham vor den Kollegen und Vorgesetzten
- ☐ Keine Wertschätzung von Kollegen für Fehlereingeständnisse
- ☐ Fehler oder ähnliches sind kein Thema für mich
- ☐ Ich habe keine Angst oder Scham und das Thema interessiert mich nicht.

Welche allgemeinen Ursachen hat Ihrer Meinung nach schlechte professionelle Kommunikation?

Mehrfachantworten möglich

- [ ] Führungsverhalten
- [ ] Arbeits- und Betriebsorganisation
- [ ] Charakterzüge der Kollegen
- [ ] Mangelnde Fortbildungsmöglichkeiten zu diesem Thema
- [ ] Es werden zu wenige Fortbildungen zu diesem Thema wahrgenommen
- [ ] Charakterzüge der eigenen Person
- [ ] Andere Ursache: ____________

Mit welcher dieser Ursachen, die zu schlechter Kommunikation führen können, wurden Sie bisher persönlich konfrontiert?

Mehrfachantworten möglich

- [ ] Führungsverhalten
- [ ] Arbeits- und Betriebsorganisation
- [ ] Charakterzüge der Kollegen
- [ ] Mangelnde Fortbildungsmöglichkeiten zu diesem Thema
- [ ] Es werden zu wenige Fortbildungen zu diesem Thema wahrgenommen
- [ ] Charakterzüge der eigenen Person
- [ ] andere Ursache: ____________

Welche Hilfe beim Thema Kommunikation würden Sie sich wünschen?

Mehrfachantworten möglich

- [ ] Ich habe keinen Bedarf
- [ ] Ein Kommunikationstraining sollte regelmäßig angeboten werden
- [ ] Dieses Thema sollte bereits Thema in der Berufsausbildung sein
- [ ] Fortbildungen zu diesem Thema sollten regelmäßig angeboten werden
- [ ] Supervisionen mit einem Mediator sollten regelmäßig angeboten werden
- [ ] Andere Hilfestellung: ______

Bitte bewerten Sie folgende Aussage:

	sehr groß	groß	gering	sehr gering
Wie groß ist Ihr aktuelles Interesse für das Thema Kommunikation?	○	○	○	○

In welchem Bundesland sind Sie tätig?

Bitte wählen... ▾

» Umleitung auf Schlussseite von Umfrage Online
