# Supplementary material for: Communication of preclinical emergency teams in critical situations: A nationwide study
Source: PLoS One. 2021 May 3;16(5):e0250932. doi: 10.1371/journal.pone.0250932 (PMC8092665; doi:10.1371/journal.pone.0250932)
Supplement: Attachment — Submitted filename: Response to reviewers_final.docx [file pone.0250932.s004.docx]

**Medizinische Klinik I**

Dr. med. Matthias Zimmer

Telefon: 069-85 05-3130 | Fax: 069-85 05-273 | zimmer.m@ketteler-krankenhaus.de | Datum: 15. Februar 2021

Unser Zeichen: MZ

Editorial team of PLOS ONE

**Response to Reviewers**

Dear Sir or Madam,

Thank you for giving us the opportunity to revise our manuscript. We have carefully considered your suggestions and have made some extensive modifications.

We have specified the description of the participants in terms of age, gender and work experience. The corresponding data are now presented in Table 1.

Statistical analyses were performed under the advice of the Institute for Biostatistics and Mathematical Modelling at the Centre for Health Sciences, Goethe-University Frankfurt. We cannot retrospectively substantiate the rationale for omitting an inferential statistic. However, we agree with your assessment and have removed the references to statistical analysis. We have restricted ourselves to a pure description of the results.

We have taken up reviewers´ suggestions and commented on the selection of items.

As had been proposed we have described in detail the procedure of invitation to take part in the study and the process of data collection. That my help readers´ perception of the study. Participants were not contacted directly because we did not know them personally. Rather, we sent general invitations to participate to the Emergency Medical Services (EMS) stations. In parallel, we asked the EMS regional medical directors for additional forwarding of the invitations to the subordinate EMS stations. Not all directors supported us. Participation was via a link to the world wide web and involved only a small investment of time for participants. There was no compensation for costs and no other contributions.

We cannot give detailed figures about response rate because it is not known how many employees in the EMS received the invitation.

At your suggestion, we have revised the content and structure of the results and discussion sections. The order of the discussion is now parallel to the presentation of the results. We prefer to present the results and the discussion separately, since our hoped-for target group is from the medical field and is strongly accustomed to this form of presentation.

Unfortunately, we did not adequately describe the context in the acknowledgement. The regional medical directors of EMS did not fund us, but authorized letters of invitation in their counties and forwarded them to the subordinate EMS stations. The Institute for Biostatistics and Mathematical Modelling at the Centre for Health Sciences, Goethe-University Frankfurt helped collegially as we are faculty members of Goethe-University Frankfurt and this is the mission of the Institute. There are no financial or other dependencies between us and the institute. We kindly ask you to reassess the facts.

Reviewer 1

We thank you for the detailed review comments and have carefully edited all points. At the same time, we have redesigned result and discussion section, respectively, to make them easier to understand. With the restriction to a purely descriptive presentation of the results and after the redesign of the results, all existing data are available to you.

We hope to have responded intensively to all your suggestions and hope for an acceptance of our article in PLOS ONE.

With best regards

Matthias Zimmer

Daria M. Czarniecki

Stefan Sahm
