# Supplementary material for: Communication of preclinical emergency teams in critical situations: A nationwide study
Source: PLoS One. 2021 May 3;16(5):e0250932. doi: 10.1371/journal.pone.0250932 (PMC8092665; doi:10.1371/journal.pone.0250932)
Supplement: Attachment — Submitted filename: Response to reviewers.docx [file pone.0250932.s005.docx]

**Medizinische Klinik I**

Dr. med. Matthias Zimmer

Telefon: 069-85 05-3130 | Fax: 069-85 05-273 | zimmer.m@ketteler-krankenhaus.de | Datum: 9. April 2021

Unser Zeichen: MZ

Editorial team of PLOS ONE

**Response to Reviewers**

Dear Sir or Madam,

Thank you for giving us the opportunity to enhance our manuscript. We have taken up all reviewers´ suggestions and have made precise corrections. In particular, we have clarified two passages in the discussion.

We thank you for the productive cooperation and hope for an acceptance of our article in PLOS ONE.

With best regards

Matthias Zimmer

Daria M. Czarniecki

Stefan Sahm
